# Work-related musculoskeletal disorders amongst undergraduate nursing students at the University of Johannesburg

**DOI:** 10.4102/hsag.v25i0.1460

**Published:** 2020-12-14

**Authors:** Malany Moodley, Fatima Ismail, Armand Kriel

**Affiliations:** 1 Department of Chiropractic, Faculty of Health Sciences, University of Johannesburg, Doornfontein, South Africa

**Keywords:** Prevalence, Nursing, Musculoskeletal disorders, Biopsychosocial factors, Lower back pain

## Abstract

**Background:**

Work-related musculoskeletal disorders (WRMSDs) present as pain or discomfort in the musculoskeletal system that individuals experience from work-related activities. Substantial research evidence exists on qualified nurses with WRMSDs, but there is a distinct lack of research regarding nursing students and their work environment in South Africa.

**Aim:**

The primary aim of this study was to establish a baseline prevalence of musculoskeletal disorders (MSDs) amongst undergraduate nursing students. The secondary aim was to identify the role of certain occupational and biopsychosocial factors in the development of MSDs.

**Setting:**

The University of Johannesburg, Health Sciences Faculty, Doornfontein Campus, Johannesburg, South Africa.

**Method:**

A cross-sectional quantitative study conducted through a questionnaire (from 24 June to 29 July 2019) was initiated amongst the undergraduate nursing students at the University of Johannesburg, South Africa. All 250 undergraduate nursing students were given the opportunity to participate by completing the Nordic Musculoskeletal Questionnaire-Extended (NMQ-E). A total of 125 questionnaires were collected and used in the study. Data analysis consisted of frequencies, descriptives and custom tables. The Chi-square test for association was used to test the associations between variables.

**Results:**

The study found an 83% prevalence of MSDs. Musculoskeletal disorders occurred most commonly in the lower back (81.1%), neck (65.9%) and shoulder (63.6%) regions. Factors that showed associations with overall MSDs were female gender (*p* = 0.002), height (*p* = 0.009) and studying at home (*p* = 0.040). Stress and tablet or smartphone use had significant associations with certain body regions.

**Conclusion:**

The findings indicate a high prevalence of MSDs in undergraduate nursing students in this sample, substantially higher than in other similar studies in which lower back disorders were most prevalent.

## Introduction

Work-related musculoskeletal disorders (WRMSDs) can be defined as any symptoms that arise from the work environment. These symptoms can be classified as impairment, discomfort, disability, persistent pain in joints, muscle, bones, ligaments and tendons, with or without physical manifestations, where the most common symptom is pain (Mohammadi 2013). These musculoskeletal disorders (MSDs) can be organised into three categories: inflammatory or non-inflammatory disorders, whether the disorder affects a specific body region and whether it is limited to the musculoskeletal system or also affects other systems in the body (Shiel 2005; Weinstein Yelin & Watkins-Castillo 2020). The National Institute for Occupational Safety and Health defines MSDs as a cluster of disorders where there is involvement of the nerves, tendons, muscles and supporting structures such as intervertebral discs (Piedrahita 2006; Woolf & Pfleger 2003).

Whilst healthcare providers are often concerned about their patients’ well-being, they may not always pay attention to their health. Several studies have been conducted that have found numerous WRMSDs in nurses (Durmus & Ilhanli 2012; Ribeiro, Serranheira & Loureiro 2017; Woolf & Pfleger 2003). The present study was conducted to investigate the prevalence of MSDs in students studying nursing. There is a global lack of research on the prevalence of MSDs in this sample group, which is surprising as these students commonly go on to develop WRMSDs once qualified (Durmus & Ilhanli 2012; Ribeiro et al. 2017; Woolf & Pfleger 2003). The inclusion of nursing students in this type of study could help the nursing students become more aware of their musculoskeletal health and attempt to prevent any future WRMSDs.

Lower back and neck pain are the most common MSDs, affecting 43.3% of the population at any given time (Woolf & Pfleger 2003). The most common MSD from which adults suffer in the workplace is reported to be lower back pain (Durmus & Ilhanli 2012; Tsekoura et al. 2017). This complaint is also the reason why most people consult a primary healthcare physician. According to the World Health Organization (WHO 2019), MSDs are the largest driver of disability throughout the world and are also the leading cause of disability at the workplace over both the short and long terms. Whilst not everyone exposed to the risk factors in the workplace will develop WRMSDs (Durmus & Ilhanli 2012), in the last 15 years, the incidence of WRMSDs has increased and become the main cause of disability before retirement. Such injuries usually occur because of moving heavy objects, repetitive physical activities, inadequate ergonomics or poor posture. Ergonomic factors in the lower back relate to incorrect or heavy lifting and frequent bending of the trunk. Disorders in the neck are often caused by twisting or bending the neck whilst twisting and elevating and abducting the shoulders for prolonged periods of time (Da Costa & Vieira 2009; Ha et al. 2009; Woolf & Pfleger 2003).

Nurses who work in hospitals are more prone to occupational health risks than other healthcare practitioners, most commonly MSDs (Chiwaridzo et al. 2018; Lipscomb et al. 2004). The most common WRMSDs found in nurses are lower back, ankle, knee, shoulder, neck, hand and wrist pain, with lower back pain being the most prevalent. This is owing to heavy load handling whilst working with patients, such as shifting patients and picking them up (Lipscomb et al. 2004; Ribeiro et al. 2017; Tinubu et al. 2010). The second most prevalent MSD amongst nurses is foot and ankle injuries. Nurses spend almost half of their time on their feet and walking between wards (Newman et al. 2014).

Work-related musculoskeletal disorders have also been shown to be the leading cause of early retirement (Brennan-Olsen et al. 2017; Ha et al. 2009). Studies have shown that appropriate ergonomic intervention is necessary to address MSDs in nurses, as well as in optometrists (Fethke et al. 2015). This study aimed to determine the prevalence of WRMSDs amongst undergraduate nursing students at the University of Johannesburg in South Africa by using a questionnaire to identify the MSDs that are most prevalent in this population. Associations between certain occupational and biopsychosocial factors and the development of MSDs in this student population were also investigated.

## Methods

### Study design and type

This study was cross-sectional, exploratory and quantitative in nature. It was conducted by distributing a questionnaire amongst undergraduate nursing students at the Doornfontein campus of the University of Johannesburg in South Africa. The study was guided by questions relating to the prevalence (overall, last 12 months and lifetime) of MSDs experienced by the undergraduate nursing students at the University of Johannesburg. The study attempted to determine which risk factors had an association with the established prevalence of MSDs in this cohort of students. The risk factors that were investigated included demographic, biographic and biopsychosocial factors, activity type as well as the level and number of hours spent on certain activities.

### Sample selection and inclusion criteria

The researchers used a convenience sampling method in this study. This sampling method was used as all the undergraduate nursing students at the University of Johannesburg were given the opportunity to participate. The total undergraduate population comprised 250 nursing students. An estimated 98 nursing students’ responses were needed to meet the minimum requirements, to allow for a 5% margin of error (Boyd, Manheim & Buhsmer 2019). All the participants selected had to be full-time University of Johannesburg undergraduate nursing students. A total of 125 questionnaires were collected, resulting in a response rate of 68%, and all were used in the study.

### Data collection

Participants were recruited at the end of stipulated classes based on prior communication with class representatives. The lecturer was not present during this process to ensure that there was no pressure on the students to complete the questionnaire. Participants received an information letter and a consent form. The researchers verbally read through the information letter and answered any questions raised by the participants. If there were no queries, the researcher handed out the questionnaires and left the venue to ensure confidentiality. Recruited participants were required to sign the information letter and the consent form specific to this study, confirming that they fully understood what was expected from them and all the procedures involved in this study. At this point, if a student did not want to participate, he or she could leave without the researchers’ knowledge. Two sealed marked boxes were placed in the venue: one marked ‘consent forms’ and the other marked ‘questionnaire’. Participants were asked to place the consent forms and completed questionnaires into the appropriate boxes. The completed surveys were collected the following day from the locked venues. Because of the large sample size and the varying timetables, a questionnaire was the most time and cost-effective method of sampling.

### Measurements and questionnaire development

The data were collected using an adapted version of the Nordic Musculoskeletal Questionnaire-Extended (NMQ-E) (Dawson et al. 2009). The Nordic and Dutch Musculoskeletal Questionnaires, according to Kuorinka et al. (1987) and Hildebrandt et al. (2001), are proven to be valid and reliable in previous studies when measuring the prevalence of MSDs. Dawson et al. (2009) found that the NMQ-E can be used as a tool to screen for musculoskeletal pain and is reliable for collecting data on musculoskeletal pain, specifically onset and prevalence. The NMQ-E was found to have a Cronbach’s alpha value ranging between 0.81 and 0.92 (Pugh et al. 2015). A statistician at the University of Johannesburg’s statistics department, Statistical Consultation Services (STATKON), assisted with the adaptation and validation of the questionnaire to ensure that it was viable for the research study. A pilot study was conducted using five participants. The data collected from the pilot study were also used in the final analysis.

The additions made by the researchers to the questionnaire, for the purposes of this study, were the inclusion of sections A (5 questions on demographic data), B (18 questions on biographical data) and C (3 questions relating to classes at the university). Sections A–C comprised 26 questions in total, whilst section D comprised 12 questions that had to be answered for each of the nine body regions. The questionnaire consisted of a nominal (yes or no) scale. Responses were excluded if 75% or less of the questions were not answered.

### Data analysis

Data analysis consisted of frequencies, descriptive statistics and custom tables. Frequencies describe categorical data in order to determine how often each specific answer was received. Descriptive statistics are made up of mean, median, mode, interquartile range, standard deviation, minimum and maximum to describe the continuous data. Customs tables were used to describe the data collected from the questions where participants can provide more than one answer. The software used for the statistical analysis was IBM Statistical Package for the Social Sciences (SPSS) version 26.0.

The chi-square test of association was used to test for any associations between variables, especially for associations between department and MSD prevalence, type of MSD, risk factors of MSD and consequences. The Cramer’s V test was used to quantify the strength of the chi-square results. Chi-square tests and Cramer’s V test were used in this study because of the need to assess associations amongst the above-stated factors. Additionally, the data collected were categorical in nature, therefore meeting the main assumptions of the tests (Warner 2013).

### Ethical considerations

Permission was requested to and granted by the Head of the Nursing Department at the University of Johannesburg to conduct the study and ethical clearance was obtained from the Health Sciences Research Ethics Committee at the University of Johannesburg (REC-01-16-2019).

The participants were informed that their participation was on a voluntary basis and that they were free to withdraw from the study at any stage until the point of submission. Thereafter, because of the anonymous nature of the study, withdrawal would have been impossible.

Each participant’s privacy was protected by ensuring anonymity and confidentiality. It was explained to all the participants that they would remain anonymous and that there was no way that the researcher would be able to track the information entered in the questionnaires back to them. In order to achieve anonymity, all the participants used a similar black ink pen and two sealed boxes were provided for collection. All the consent forms were placed in one box and the questionnaires were placed in the other to separate the participants’ information from their answers. The researcher and lecturer vacated the classroom whilst the participants completed the questionnaires. Once completed, the participants placed their own forms in the appropriate boxes.

There were no risks to or direct benefits for the participants. The research consisted of a questionnaire that was completed by the participants in the classrooms after their lectures. The future benefits include knowledge of WRMSDs and, more broadly, the development of preventative measures on how to avoid such disorders.

## Results

### Responses

The total student population in the undergraduate nursing department at the University of Johannesburg comprises 250 students. Ninety-eight student responses from a possible 250 were needed to allow for a 5% margin of error (Boyd et al. 2019). We received a total of 125 responses (with a response rate of 68%), which surpassed the minimum amount of responses needed. Possible reasons for non-participation included lack of time, students not being present on the day the survey was conducted, or refusal to participate.

### Baseline characteristics

#### Gender, age, height and weight

Of the undergraduate nursing students who comprised this sample (*n* = 125), 77.6% (*n* = 97) were women and 22.4% (*n* = 28) were men (Table 1). Female students complained of MSDs more than male students (*p* = 0.002). Female participants had significantly more neck (*p* = 0.001), upper back (0.018) and lower back (0.001) musculoskeletal complaints than their male counterparts.

**TABLE 1:** Demographic data.

Demographic	*n*	*m*	kg	years
**Gender**				
Male	28	-	-	-
Female	97	-	-	-
**Total**	**125**	**-**	**-**	**-**
**Age**				
Mean	-	-	-	22.36
**Height**				
Mean	-	1.56	-	-
**Weight**				
Mean	-	-	66.13	-

The mean age of the sample (Table 1) was 22.36 years (standard deviation [SD] = 2.638), the mean height was 1.56 m (SD = 0.138) and the mean weight was 66.13 kg (SD = 12.997). Height seemed to have an influence on the prevalence of MSDs (*p* = 0.009). Age and weight did not show any statistical significance to the prevalence of MSDs.

#### Year of undergraduate study

Most of the students who comprised this sample (Table 2) were in their 4th year (42.7%, *n* = 53), whilst 29.8% (*n* = 37) were in their third year, 25.8% (*n* = 32) were in their second year and 1.6% (*n* = 2) were in their first year of study. The year that students were in did not show any statistical significance in the overall prevalence of MSDs.

**TABLE 2:** Year of undergraduate study.

Year	*n*	%
Fourth	53	42.7
Third	37	29.8
Second	32	25.8
First	2	1.6

### Prevalence of musculoskeletal disorders in nursing students

#### The overall prevalence of musculoskeletal disorders amongst the undergraduate student cohort in the nursing department

There was an 83% prevalence of MSDs in the total sample of undergraduate nursing students. Of this, 82.7% (*n* = 86) were represented by female students whilst 17.3% (*n* = 18) were represented by male students.

#### Prevalence of musculoskeletal disorders in the last 12 months

Table 3 reports the prevalence of MSDs experienced by undergraduate nursing students, both in the last 12 months and in their lifetime. In the last 12 months, 81.1% (*n* = 71) of the sample population had suffered from lower back pain, 65.9% (*n* = 58) had neck pain, 63.6% (*n* = 49) had shoulder pain, 63.6% (*n* = 49) had ankle and foot pain and 62.2% (*n* = 42) had upper back pain. The body regions least affected included the elbow (11.1%, *n* = 6), hand and wrist (41.5%, *n* = 27) and hip and thigh (46.8%, *n* = 29) regions.

**TABLE 3:** Prevalence of musculoskeletal disorders in nursing students (lifetime and the last 12 months).

Body region	Lifetime prevalence of MSD disorders		Prevalence of MSD disorders in the last 12 months	
	*n*	%	*n*	%
Neck	78	66.7	58	65.9
Shoulders	69	60.0	49	63.6
Upper back	59	51.3	46	62.2
Elbows	9	8.2	61	1.1
Hand and wrist	37	33.3	27	41.5
Low back	86	74.8	73	81.1
Hips and thighs	36	32.7	29	46.8
Ankles and feet	65	56.5	49	63.6

MSD, musculoskeletal disorders.

#### The lifetime prevalence of musculoskeletal disorders

Over their lifetime, 78.8% of students reported suffering from lower back pain (*n* = 86), 66.7% (*n* = 78) from neck pain, 60.0% (*n* = 69) from shoulder pain, 56.5% (*n* = 65) from ankle and foot pain and 51.3% (*n* = 59) from upper back pain. The body regions least affected included the hand and wrist (33.3%, *n* = 37), hip and thigh (32.7%, *n* = 36) and elbow (8.2%, *n* = 9) regions.

#### Health practitioners consulted for the musculoskeletal disorders

A general practitioner (72.7%, *n* = 8) was most frequently consulted for treatment of the various MSDs. Chiropractors and physiotherapists were seen by 18.2% (*n* = 2), respectively. ‘Other’ forms of treatment were sought by 27.3% (*n* = 3) of students. Homoeopaths, bio-kineticists and surgeons were seen by 9.1% (*n* = 1), respectively. Of the 125, only 8.8% (*n* = 11) of students answered this question.

### Hours spent per week doing various tasks

#### Number of hours spent per week sitting in the class

Fifty-nine (47.2%) nursing students spent under 10 h per week sitting in the class. Just over one-third (33.6%; *n* = 42) of students spent between 10 and 20 h per week sitting in the class. Students that spent between 20 and 30 h per week sitting in the class constituted 17.6% (*n* = 22) of the sample, whilst only 1.6% (*n* = 2) of students spent more than 30 h per week sitting in the class. Time spent sitting in the class showed a statistically significant effect on the prevalence of upper back musculoskeletal complaints (*p* = 0.028).

#### Number of hours spent per week in practical classes

Table 4 indicates that the majority of nursing students (63.4%, *n* = 78) spent between 16 and 20 h per week in practical classes, whilst 15.4% (*n* = 19) spent between 6 and 10 h per week in practical classes. The amount of time spent in practical classes did not have an effect on the prevalence of MSDs. Students believed that their overall musculoskeletal pain (*p* = 0.000), and specifically their neck (*p* = 0.002), upper back (*p* = 0.004) and lower back (*p* = 0.004) musculoskeletal pain, was related to university practical classes.

**TABLE 4:** Number of hours, per week, spent in practical classes.

Number of hours spent per week in practical classes	Number of nursing students	
	*n*	%
0–5 h	12	9.8
6–10 h	19	15.4
11–15 h	14	11.4
16–20 h	78	63.4

**Total**	**123**	**98.4**

#### Number of hours spent per week at home studying

Thirty-five per cent (*n* = 44) of students spent only 0–5 h at home studying per week, whilst 33.6% (*n* = 42) students spent between 6 and 10 h at home studying per week. Students who spent 11–15 h of studying at home comprised 23.2% (*n* = 29) of the sample. Ten students (8%) spent between 16 and 20 h at home studying. The prevalence of MSDs in the last 12 months showed a statistically significant value in relation to the time spent studying at home (*p* = 0.040).

#### Number of hours spent per week on computer usage

Table 5 indicates that most of the students (53.6%, *n* = 67) spent about 0–5 h per week using a computer. Twenty-seven per cent (*n* = 34) of the sample spent 6–10 h on computer usage per week. Time spent at the computer did not yield any statistically significant results.

**TABLE 5:** Number of hours spent per week on computer usage.

Number of hours spent per week on computer usage	Number of nursing students	
	*n*	%
0–5 h	67	53.6
6–10 h	34	27.2
11–15 h	12	9.6
16–20 h	12	9.6

**Total**	**125**	**100**

#### The intensity of cell phone or tablet usage for personal and university purposes

As shown in Table 6, students reported very high usage (52.8%, *n* = 68) and high usage (32%, *n* = 40) of cellular phones and tablets per week. Intensity of cell phone and tablet usage showed statistically significant values for the prevalence of neck (*p* = 0.045) musculoskeletal complaints.

**TABLE 6:** Intensity of cell phone or tablet usage for personal and university purposes.

Intensity of cellular phone or tablet use for both private and university purposes	Number of nursing students	
	*n*	%
Low use	1	0.8
Moderate use	18	14.4
High use	40	32.0
Very high use	66	52.8

### Activities during practical classes

#### Movements most performed during practical classes

Most students spent time in practical classes standing (37.6%, *n* = 47) and walking (36.0%, *n* = 35). Thereafter, 21.6% (*n* = 27) of the time was spent on sitting. Only 4.8% (*n* = 6) of students reported bending over in practical classes. Performing repetitive movements (*p* = 0.004) in the class showed a statistical significance in relation to the prevalence of upper back complaints.

#### The activity most performed in practical classes

In this study, 83.9% (*n* = 104) of the students reported that they spent their practical classes in moving equipment around, whilst 47.6% (*n* = 59) of the students stated that they spent their time in moving patients around. Treating patients only comprised 27.4% (*n* = 34) of students’ time. Students indicated that they used their backs (52.5%) when lifting patients and heavy equipment instead of their knees (15.6%). This showed a statistical significance with upper back (*p* = 0.004) and lower back (*p* = 0.014) musculoskeletal complaints.

### Biopsychosocial factors experienced by nursing students

#### The intensity of students’ stress-level this year

Nursing students indicated that they experienced high levels of stress. Very high stress levels were reported by 31.5% (*n* = 39) of the students. High (33.9%, *n* = 42) and moderate (30.6%, *n* = 38) stress levels were also reported. Very low and low levels of stress were experienced by only 4% (*n* = 5) of the students. The intensity of stress had a statistically significant effect on the prevalence of upper back (*p* = 0.006) and lower back (*p* = 0.000) MSDs.

#### Number of hours spent per week on exercise

An equal number of students reported spending no time (41.6%, *n* = 52) or 0–3 h (41.6%, *n* = 52) on exercise per week. Thereafter, 8.8% (*n* = 11) of students spent between 4 and 6 h on exercise, 6% (*n* = 6) spent 7–9 h, 3% (*n* = 3) spent 10–12 h and only 0.8% (*n* = 1) spent more than 13 h on exercise per week. Walking showed a statistically significant value in relation to the prevalence of both neck (*p* = 0.034) and lower back (*p* = 0.032) MSD complaints.

In conclusion to the results, it is distinguished that factors that have an influence on the prevalence of MSDs in the nursing students include gender, height, whether studying at home, tablet or cell phone usage, using the back (instead of the knees) for repetitive movements and heavy lifting, stress and physical activity, specifically walking. Students also believed that their musculoskeletal pain is related to university practical classes.

## Discussion

This is the first study to determine the prevalence of MSDs amongst undergraduate nursing students enrolled at the University of Johannesburg in South Africa. The overall prevalence of MSDs in the sample (83%) was surprisingly high, demonstrating both conflicting and similar percentages to other comparative studies. Munabi and Madadzedah (2014) found an 80% prevalence of MSDs amongst medical and nursing professionals at two sampled Ugandan university hospitals. Professional nurses are said to be highly exposed to MSDs as a result of the nature of their work. The relatively high MSDs prevalence amongst nursing professionals has been discussed as a norm by Tinubu et al. (2010) and Ribeiro et al. (2017).

Abledu and Offei (2015) found a comparatively lower prevalence rate amongst nursing students at a Ghanaian college (70.1%), whilst Panebianco (2017) found an 83% prevalence of MSD amongst music students at the University of Pretoria. Almhdawi and Choobineh (2017) asserted that the worldwide MSD prevalence is 67.1%. These authors compared this finding with the prevalence of MSDs amongst Brazilian nursing students (87%). The findings of the current study, together with those from Almhdawi and Choobineh (2017), point towards a significantly high MSD prevalence amongst nursing students, for which intervention may be critical in ensuring the overall future well-being of healthcare workers.

The sample of nursing students of the present study showed a high pain and discomfort prevalence in most body regions. The most affected body regions with the highest prevalence rates in the last 12 months included the lower back (81.8%), neck (65.9%), shoulder, ankle and foot (63.6%), followed by upper back (62.2%). When compared to the studies by Munabi and Madadzedah (2014) on health sciences students and Morais et al. (2019) on nursing students, it is apparent that their prevalence rates were lower than what was seen in the current study. A study by Moodley and Naidoo (2015) on the prevalence of MSD on dentistry students was more in line with our findings regarding high MSD prevalence. However, a common trend amongst all the studies (Moodley & Naidoo 2015; Morais et al. 2019; Munabi & Madadzedah 2014), including the present study, is that lower back pain is the most frequently occurring MSD.

Lower back pain has been investigated and identified as being commonly associated with occupational, organisational, lifestyle and psychosocial factors amongst working professionals in general (Alghwiri & Marchetti 2018; Balakrishnan & Chellappan 2016). It was therefore not surprising to find that disorders in this region were the most prominent amongst the sampled health students. Other common areas affected included the neck and shoulder regions. This finding can be attributed to the occupational factors associated with medical disciplines, including lifting, moving and shifting activities (Lipscomb et al. 2004).

Numerous studies similar to the present study have found an inclination for women to have an increased prevalence of MSDs over men and across disciplines (Jellad et al. 2013; Ng, Hayes & Polster 2016; Rodríguez-Romero et al. 2016). This was also true for our study. However, it must be taken into consideration that men are under-represented in the nursing profession (Jellad et al. 2013), as well as in this sample group; thus, gender differences are difficult to assess (Long, Johnston & Bogossian 2012). A cross-sectional descriptive study was conducted on 158 students in two male and female schools in Ardebil. A significant relationship between height and musculoskeletal features was seen (Firouz et al. 2018). In a study by Yue, Liu and Li (2012), which examined the association between demographic factors (including age, height and body mass index) and the prevalence of musculoskeletal pain, a significant relationship between the above factors and the incidence of musculoskeletal pain in both genders was also observed. Our study comparatively showed the same significance regarding height. Age did not seem to have an association with an increased prevalence of MSD in this sample. This could be because of the fact that the sample comprised students who are generally younger in age.

Dachs et al. (2014) found that 91% of medical students in South Africa were not competent in managing MSDs. This finding is concerning considering that students mostly see a general practitioner for their MSDs, even though the general public notion of chiropractors is that they are specialists of both spinal and musculoskeletal conditions (Schneider, Murphy & Hartvigsen 2016). Humphreys et al. (2007) established that senior chiropractic students were competent in the management of MSDs, which was not the case with fellow medical and physical therapy students. It is intriguing that nursing students would seek care for MSDs from general practitioners rather than chiropractors. More importantly, only 8.8% of the participants in this study sought medical care for their MSDs, which is surprising given the high prevalence of MSDs (83%). Awareness of MSD treatment options available seems questionable and shows the capacity for improvement.

In the literature, posture, repetitive movements, time spent on studying, time spent on the computer and lifting of both equipment and patients were found to have positive associations or relationships with MSDs. Studies by Panebianco (2017), Moodley and Naidoo (2015) and Morais et al. (2019) confirmed the association between the above factors and MSD prevalence. High frequencies of postural and lifting challenges were recorded in the present study, corroborating the above findings.

Morais et al. (2019) also found a strong association between the number of hours spent in the class and the prevalence of MSDs amongst health students in Brazil. Students who spent long hours sitting in the class had a comparatively higher prevalence of MSDs than those who spent fewer hours sitting in the class, with all other variables being held constant. Practical courses were found to expose students to many rigorous postural movements and lifting activities and accounted for over 80% of the MSD prevalence in the studied sample (Morais et al. 2019). These findings were analogous to those in the present study, which show that long hours spent in classes could be a possible cause of the high MSD prevalence. Nursing students, together with speech-language pathology and occupational therapy students, have a higher prevalence of MSD when compared to students from other disciplines, as they are inclined to perform activities both in in-hospital care and out. These activities assume static and non-ergonomic postures resulting in muscle overload, which can cause muscular burden (Martins & Felli 2013).

Grzywacz and Bass (2003), Piedrahita (2006) and Morais et al. (2019) stated that participants who reported high work or academic stress levels often reported suffering from MSDs, indicating that stress as a psychosocial factor was also an MSD risk factor. The findings of the current study show that 68% of the students indicated that they were either highly stressed or very highly stressed, and that stress had a statistically significant influence on the prevalence of MSDs. These high stress levels could be a contributing factor to the high overall prevalence rate of MSD in the student sample. Abledu and Offei (2015) asserted that stress levels were often higher during examination times amongst healthcare students, with these students reporting higher MSD prevalence.

Based on available data, Kelly et al. (2018) confirmed that exercise is effective in the management of work-related upper limb disorders and can promote healthier lifestyles. Resistance training in the work environment has been shown to decrease pain in body regions, such as the shoulder, wrists and cervical, dorsal and lumbar spine (Rodrigues et al. 2014). The sample in this study reported that they did very little exercise, which may have a subsequent effect on the risk of MSDs. Our sample unexpectedly showed a correlation between walking and the incidence of neck and lower back MSDs. This is contrary to other studies that have shown that physical activity such as walking decreases the risk of MSDs (Hendi et al. 2019; Kelly et al. 2018; Rodrigues et al. 2014).

According to two independent reviews presented by Eitivipart, Viriyarojanakul and Redhead (2018) and Xie, Szeto and Jie (2016), it was found that the use of smartphones induced musculoskeletal symptoms specifically in the neck region. Another study by Sharan et al. (2014) concluded that the use of mobile phones was associated with a higher prevalence of MSDs. Our study on nursing students showed similar associations.

## Conclusion

There is a high prevalence of MSDs (83%) in undergraduate nursing students at the University of Johannesburg, South Africa. These findings are substantially higher when compared to other prevalence studies on students and health professions from other disciplines. Lower back disorders presented with the highest prevalence rate in the last 12 months, and over the participants’ lifetime. There are also numerous factors that play a role in the prevalence of these MSDs. Given the high rates of WRMSDs in qualified nurses, it is our recommendation that students should be introduced to educational interventions to prevent and manage work-related MSDs, to prevent a contagion effect.

### Recommendations of the study

Based on the conclusions made above, the following recommendations are made. Ergonomic support for the classroom environment is recommended to reduce MSDs emanating from sitting or standing too long in the class. More frequent breaks are also recommended as a way of reducing muscle strain from sitting and standing for long periods of time in the classroom. Students should be educated on the need to visit appropriate medical professionals when experiencing MSDs; doing so will reduce the risk of MSDs becoming worse, to the point where they can cause major injuries and disabilities. Awareness should be encouraged regarding the biopsychosocial well-being of students, with possible implementations of exercise protocols and stress management programmes. Students should be educated on less precarious sitting and working postures, including those for lifting and carrying patients and items related to work and study.

### Limitations of the study

Some limitations that were noted in the process of carrying out the study included the following: the study relied on self-assessments or self-ratings of students’ experiences and perceptions of MSDs. There is a risk that some students might not have been able to effectively recall all their experiences and perceptions, leading to less reliable answers to some questions. The study used a sample exclusively from the University of Johannesburg. It cannot therefore guarantee the generalisability of the findings to students attending other universities and health courses in South Africa.

### Recommendations for future studies

The following studies are recommended as a way of enhancing the findings on the prevalence of MSDs amongst university students in South Africa. Future studies should identify the factors that determine the choice of the type of medical practitioners to approach for MSD treatment and management. There should be an assessment of MSD prevalence amongst other South African university students in general.
